# Rich microbial and depolymerising diversity in Antarctic krill gut

**DOI:** 10.1128/spectrum.04035-23

**Published:** 2024-03-11

**Authors:** Lars Möller, Yevhen Vainshtein, Bettina Meyer, John Neidhardt, A. Murat Eren, Kai Sohn, Ralf Rabus

**Affiliations:** 1 Institute for Chemistry and Biology of the Marine Environment (ICBM), Carl von Ossietzky Universität Oldenburg, Oldenburg, Germany; 2 In Vitro Diagnostics, Fraunhofer Institute for Interfacial Engineering and Biotechnology (IGB), Stuttgart, Germany; 3 Helmholtz Institute for Functional Marine Biodiversity at the University Oldenburg (HIFMB), Oldenburg, Germany; 4 Biosciences, Alfred Wegener Institute (AWI), Helmholtz Centre for Polar and Marine Research, Bremerhaven, Germany; 5 Department of Human Medicine, Carl von Ossietzky Universität Oldenburg, Oldenburg, Germany; 6 Research Center Neurosensory Science, Carl von Ossietzky Universität Oldenburg, Oldenburg, Germany; 7 HIFMB-MPG Bridging Group for Marine Genomics, Max Planck Institute for Marine Microbiology, Bremen, Germany; 8 Alfred Wegener Institute (AWI), Helmholtz Center for Polar and Marine Research, Bremerhaven, Germany; Panepistemio Thessalias Tmema Geoponias Ichthyologias kai Ydatinou Periballontos, Volos, Greece

**Keywords:** gut microbiome, Antarctic krill, *Euphausia superba*, cultivation, metagenome, phylogeny, hydrolytic enzymes, biopolymers

## Abstract

With almost a quadrillion individuals, the Antarctic krill processes five million tons of organic carbon every day during austral summer. This high carbon flux requires a broad range of hydrolytic enzymes to decompose the diverse food-derived biopolymers. While krill itself possesses numerous such enzymes, it is unclear, to what extent the endogenous microbiota contribute to the hydrolytic potential of the gut environment. Here we applied amplicon sequencing, shotgun metagenomics, cultivation, and physiological assays to characterize the krill gut microbiota. The broad bacterial diversity (273 families, 919 genera, and 2,309 species) also included a complex potentially anaerobic sub-community. Plate-based assays with 198 isolated pure cultures revealed widespread capacities to utilize lipids (e.g., tributyrin), followed by proteins (casein) and to a lesser extent by polysaccharides (e.g., alginate and chitin). While most isolates affiliated with the genera *Pseudoalteromonas* and *Psychrobacter*, also *Rubritalea* spp. (Verrucomicrobia) were observed. The krill gut microbiota growing on marine broth agar plates possess 13,012 predicted hydrolyses; 15-fold more than previously predicted from a transcriptome-proteome compendium of krill. Cultivation-independent and -dependent approaches indicated members of the families *Flavobacteriaceae* and *Pseudoalteromonadaceae* to dominate the capacities for lipid/protein hydrolysis and to provide a plethora of carbohydrate-active enzymes, sulfatases, and laminarin- or porphyrin-depolymerizing hydrolases. Notably, also the potential to hydrolyze plastics such as polyethylene terephthalate and polylactatide was observed, affiliating mostly with Moraxellaceae. Overall, this study shows extensive microbial diversity in the krill gut, and suggests that the microbiota likely play a significant role in the nutrient acquisition of the krill by enriching its hydrolytic enzyme repertoire.

**IMPORTANCE:**

The Antarctic krill (*Euphausia superba*) is a keystone species of the Antarctic marine food web, connecting the productivity of phyto- and zooplankton with the nutrition of the higher trophic levels. Accordingly, krill significantly contributes to biomass turnover, requiring the decomposition of seasonally varying plankton-derived biopolymers. This study highlights the likely role of the krill gut microbiota in this ecosystem function by revealing the great number of diverse hydrolases that microbes contribute to the krill gut environment. The here resolved repertoire of hydrolytic enzymes could contribute to the overall nutritional resilience of krill and to the general organic matter cycling under changing environmental conditions in the Antarctic sea water. Furthermore, the krill gut microbiome could serve as a valuable resource of cold-adapted hydrolytic enzymes for diverse biotechnological applications.

## INTRODUCTION

The Antarctic krill (*Euphausia superba* Dana, in the following termed krill) represents a keystone species in the Southern Ocean ecosystem by acting as the main trophic link between primary producers and apex predators as well as by impacting biogeochemical cycles via a high production of large, carbon-rich and fast sinking fecal pellets (1). The circumpolar, panmictic population of krill comprises up to 8 × 10^14^ individuals with an approximate cumulative biomass of 3.8 × 10^8^ tons, which is comparable to the weight of humanity (2, 3). As an organism of high-energy throughput during the productive season in the Southern Ocean (summer), krill has an estimated daily food uptake of 8.5‒28% of its own body weight carbon (4–6). This equals to an average 4.7 × 10^6^ tons of organic carbon, processed every day by the global krill population via a combined gut volume of approx. 8 × 10^7^ m^3^. Accordingly, the diverse food spectrum of the omnivorous krill consists of diatoms, flagellates, lithogenic particles, protozoans, and other copepods in varying proportions and depending on the oceanic region, season, and live stage (7). In addition, the contribution of fecal pellets to carbon flux accounts for 17‒72% of the total carbon flux in highly productive regions such as the Antarctic Peninsula (8) and the marginal ice zone (9).

To effectively utilize the ingested food, the krill gut environment requires a broad spectrum of hydrolases that can decompose biomacromolecules such as lipids, proteins, and various carbohydrates (e.g., chitin or alginate). The Antarctic krill possesses a collection of highly active hydrolases encoded in its own genome (10). However, similar to other krill species (11), its gut microbiome likely has a role in the (efficient) digestion of complex biomolecular structures (12). Indeed, compared to the surrounding Antarctic waters, the krill gut harbors a 10^4^ times higher microbial abundance (12, 13), displays six times higher frequency of cell division (12), and contains distinct microbial assemblages (14, 15). This enrichment may not solely arise from filtration and uptake of accordant bacteria by the krill itself, but rather indicates the presence of an endogenous krill gut microbiome. By providing means to further process food-derived biomacromolecules, the extracellular hydrolases encoded by the endogenous krill gut microbiota may generate additional, readily absorbable monomeric nutrients that are otherwise inaccessible to the host. However, the poorly understood hydrolytic capacity of the krill gut microbiota prevents further insights into the potential impact of the changing nutrient composition and temperatures of the oceans on the ecology and population dynamics of krill and its downstream implications on the marine food web. Furthermore, the krill gut microbiota represent an untapped reservoir for the discovery of cold- and high-salinity-adapted hydrolases benefitting resource-saving biotechnological applications.

Here, we used molecular surveys and physiological assays of the Antarctic krill gut microbiota integrating cultivation-independent and -dependent approaches to improve our understanding of the microbial diversity in the krill gut and its potential to hydrolyse ecosystem-relevant biomacromolecules as well as plastics. Our findings suggest that the krill gut harbors a richer microbial diversity and a broader repertoire of depolymerizing hydrolases than previously assumed.

## RESULTS AND DISCUSSION

**Fig 1:**
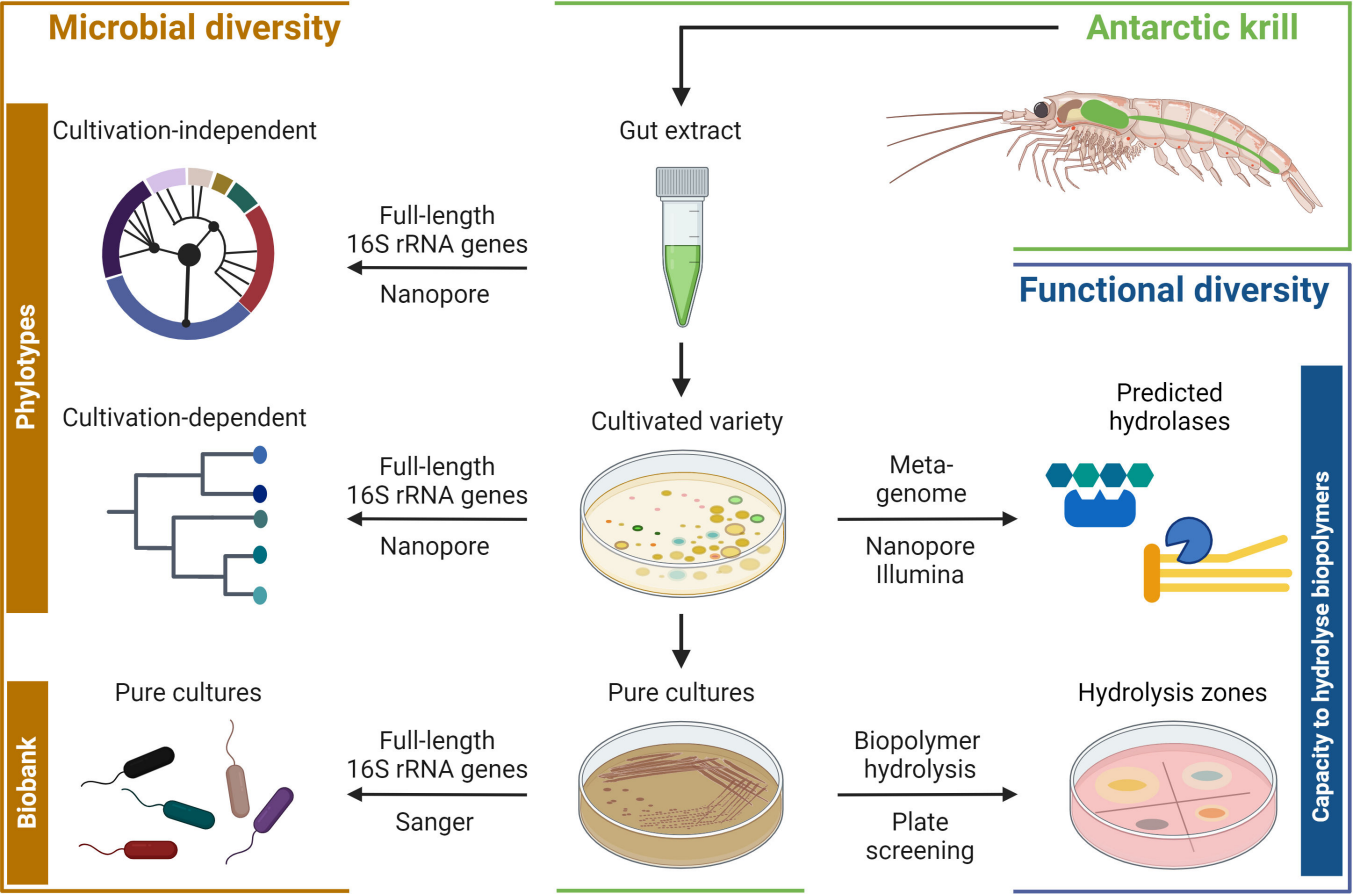
Schematic illustration of the study design comprising four major parts. First, guts were excised on shipboard from freshly harvested adult Antarctic krill (*Euphausia superba*), conserved and later extracted in the laboratory. Second, gut extracts were used to assess the cultivated variety and to isolate pure cultures. Third, microbial diversity was analyzed directly from the gut extract as well as from the cultivated variety and the pure cultures. Fourth, the potential capacities to hydrolyze (bio)polymers were investigated by metagenomic analyses of the cultivated variety as well as by plate-based assaying of the pure cultures. Applied sequencing and physiological approaches are indicated in the arrows. Created with BioRender.com.

To explore the microbial and functional diversity of the Antarctic krill gut microbiome the following study design was applied. Initially, the krill gut was dissected from freshly caught adult krill in the Bransfield Strait and conserved in glycerol solution for further analysis at the University of Oldenburg. Gut samples were extracted in the laboratory, spread on MB agar plates to access the cultivatable variety and obtain pure cultures (Fig. 1, central panel). Microbial diversity was studied by determining full-length 16S rRNA gene sequences directly from the gut extract and cultivated variety, as well as from isolated pure cultures (Fig. 1, left panel). Finally, functional diversity, namely the potential of (bio)polymer hydrolysis was studied by metagenomic analysis of cultivated variety and by plate-based assays of the isolated pure cultures (Fig. 1, right panel).

### Microbial diversity of the krill gut microbiome

**Fig 2:**
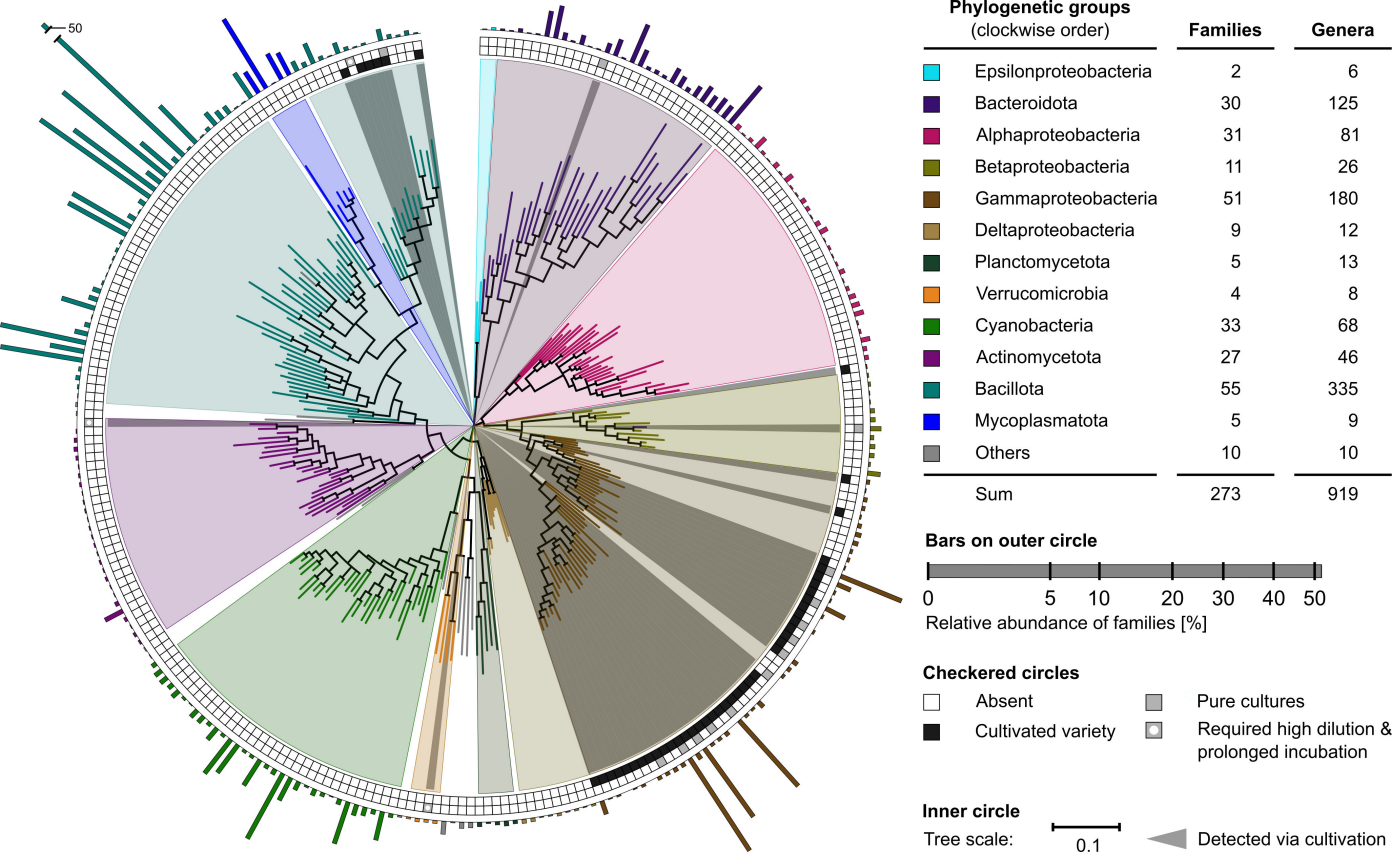
Microbial diversity (bacterial family level) of krill gut extract, cultivated variety, and pure cultures. Inner circle: the maximum likelihood phylogenetic tree is based on full-length 16S rRNA gene sequences of the most abundant bacterial species within each family, applying 1,000 iterations. Color-coding differentiates phylogenetic groups (background) and family-representing species (branches); see top right panel. Gray background highlights molecular phylotypes, which could be covered by cultivation. Checkered circles: cultivation coverage according to cultivated variety, pure cultures, and requirement for prolonged incubation; black and gray versus white boxes indicate successful versus failed cultivation. Outer circle: relative abundance of detected families based on all sequence reads assigned in each case. Top right panel: diversity of families and genera detected per phylogenetic group.

Diversity of the krill gut microbiome was analyzed based on full-length 16S rRNA gene sequences determined for the gut extract and cultivated variety by nanopore sequencing and for the isolated pure cultures by Sanger sequencing (Fig. 2). Combined results revealed a total number of 37 classes, 96 orders, 273 families, and 919 genera together comprising 2,309 species. Previous investigations based on partial-length 16S rRNA gene sequences (Illumina MiSeq) revealed 45 OTUs in the digestive gland, 137 OTUs in the stomach, and 479 OTUs in the moult microbiome of krill (14, 15). The present study revealed the five most diverse (sub)phyla (decreasing order): Bacillota, Gammaproteobacteria, Bacteriodota, Alphaproteobacteria, and Cyanobacteria. These groups were previously also detected in krill’s digestive gland and stomach (15). Despite the geographically distant locations of the present study (Bransfield Strait) and the reports by Clark et al. (14, 15) (Indian sector of the Southern Ocean), the (sub)phylum level diversity is relatively similar.

The aforementioned five bacterial (sub)phyla, as well as the low diverse Mycoplasmatota, also represent the most abundant phylogenetic groups in the here studied krill gut extracts. This agrees with earlier reports on the abundance patterns within the gut microbiome of other members of the subphylum Crustacea, e.g., horseshoe crabs (*Tachypleus tridentatus* and *Carcinoscorpius rotundicauda*) (16), hydrothermal vent crab (*Austinograea* sp.), and shallow water crabs (*Eriocheir siensis* and *Portunus trituberculatus*) (17).

A striking feature of the here resolved krill gut microbiome was the relatively high abundance of (obligate) anaerobic microorganisms affiliating with the phylum Bacillota (75% of total reads), such as typical intestinal tract inhabitants from the families *Clostridiaceae*, *Lachnospiraceae*, *Peptostreptococcaceae*, and *Oscillospiraceae* [e.g., reference (18)]. This could indicate a high food load in the gut, which results in high mineralization rates establishing anoxic conditions due to O_2_-depletion by aerobic microbes. Such a scenario was previously shown for the gut of the smaller-sized Arctic copepode *Calanus* spp. (19). Furthermore, members of these bacterial families are known for their capacity to hydrolyze food-derived biopolymers. In a previous study by Clark et al. (14), Bacillota cumulatively accounted for only 2–4% of the total reads, implicating a different state of feeding and/or gut condition. At present, seasonal effects and/or technical biases on the determined abundances of Bacillota between these two studies cannot be assessed conclusively. Moreover, the effects of diurnal cycle on the gut microbiome composition, as reported for the Chinese mitten crab (*Eriocheir sinensis*) (20), may also have to be considered.

Gammaproteobacteria may provide stable members of the krill gut microbiome or just pass to a certain extent through it, since several of the detected species have previously been isolated from ambient environments such as *Colwellia maris* from the open sea (21), *Shewanella vesiculosa* from Antarctic coastal sediments (22), and *Psychrobacter nivimaris* from Antarctic waters (23).

Members of the Mycoplasmatota represent an enriched microbial group in the krill gut (Fig. 2), which is in accord with previous observations (14). Indeed, two *Spiroplasma* spp. belonged to the 30 most abundant species observed in the present study. In the case of the deep-sea isopod *Bahynomus* sp., Mycoplasmatota symbionts were suggested to contribute to the host nutrition by proteolysis and oligosaccharide degradation (24). However, since Mycoplasmatota are known to also comprise pathogens of Crustacae representatives [e.g., reference (25)], presently, it cannot be defined whether the here detected Mycoplasmatota play a symbiotic or pathogenic role in the krill gut.

### Pure culture diversity

The aforementioned molecular diversity analyses were complemented by cultivations on MB plates, which were recently shown to allow access to a broader range of microbial diversity compared to low-complexity media (26). Here, we differentiated between cultivated variety (enrichment) and isolated pure cultures (according to morphological distinction) (Fig. 2, checkered outer circles). In total, 198 isolates from krill gut extract were obtained, which could be assigned to the five (sub)phyla Bacteriodota, Bacillota, Actinomycetota, Verrucomicrobia, and Gammaproteobacteria. While these were also detected with the cultivation-independent approaches, they represent less than 50% of the sub(phyla) resolved overall by the latter. The observed reduced diversity aligns with prior research that employed complex media to investigate cultivatable microbial diversity within the nutrient-rich krill gut (27–29). This may simply reflect the selection for copiotrophic bacteria adapted to nutrient-rich marine habitats, out-competing their oligotrophic counterparts (30–32). Such a scenario is corroborated by the more refined inspection of the obtained isolates on the genus-/species-level, as detailed in the following.

The vast majority of isolates (92%) are affiliated with the subphylum Gammaproteobacteria, distributed across 11 families and 12 genera therein. Genera with the highest number of assigned isolates were (decreasing order): *Psychrobacter* (family *Moraxellaceae*), *Pseudoalteromonas* (family *Pseudoalteromonadaceae*), *Shewanella* (family *Shewanellaceae*), and *Colwellia* (family *Colwelliaceae*). These four genera are known to encompass typical copiotrophic bacteria [e.g., reference (31)] and were also detected as the most highly abundant gammaproteobacterial hits in the present 16S rRNA gene analysis (Fig. 2). Dominance of the genus *Psychrobacter* was previously also observed in cultivation-dependent studies on the krill gut (27–29). Noteworthy, several isolates of the present study have not been described for the krill gut before, e.g., *Oleispira antarctica*, which was previously obtained from Antarctic coastal sea water (33).

The second most common group of isolates (5%) is affiliated with the phylum Bacteriodota, distributed across four genera. Most of the isolates belonged to the genus *Bizionia*, members of which occur in diverse marine habitats, including sea water from oyster farms (34) and the intestinal tract of the ubiquitous, commercially relevant fish splendid alfonsino (*Beryx splendens*) (35). While our cultivation-independent approach (Fig. 2) revealed the families *Clostridiaceae*, *Lachnospiraceae*, *Peptostreptococcaceae*, and *Oscillospiraceae* as abundant constituents of the krill gut microbiome, they evaded the current cultivation attempts due to their (obligate) anaerobic lifestyle.

Rare representatives of the isolates obtained in this study include close relatives of *Dietzia psychralcaliphila* (phylum Actinomycetota), isolated from Deception Island (Bransfield Strait) (36), and of *Rubritalea profundi* (phylum Verrucomicrobia), isolated from deep-sea water of the Pacific Ocean (37).

### Biopolymer-hydrolysing potential of pure cultures

**Fig 3:**
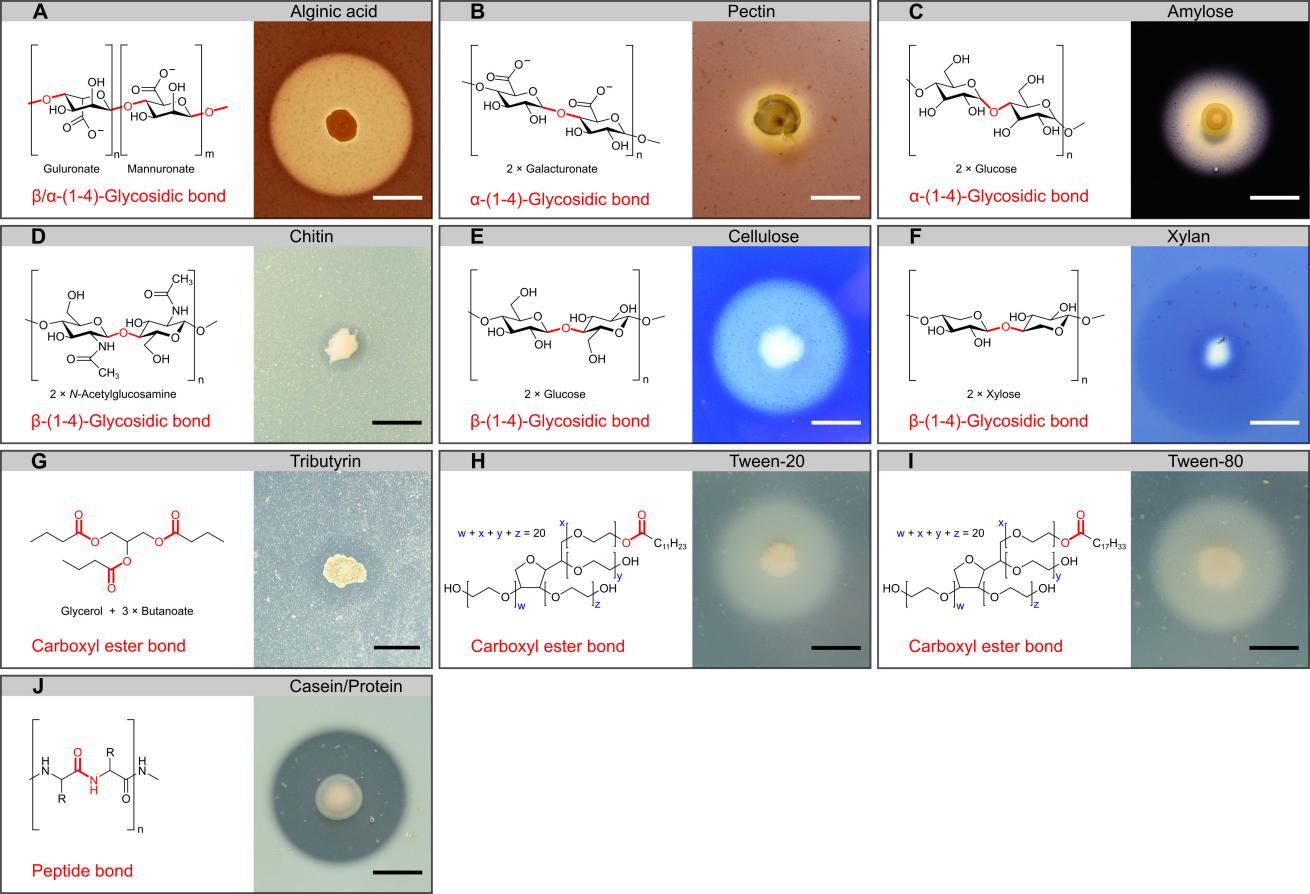
Plate-based biopolymer degradation assay. Per biopolymer tested, targeted chemical bonds (left part) are schemed and hydrolysis zones on the plates (right part) are exemplified. Bar at each hydrolysis zone represents 10 mm. The tested biopolymers included six polysaccharides (A−F), three lipid-like structures (G−I), and one protein mixture (**J**).

**Fig 4:**
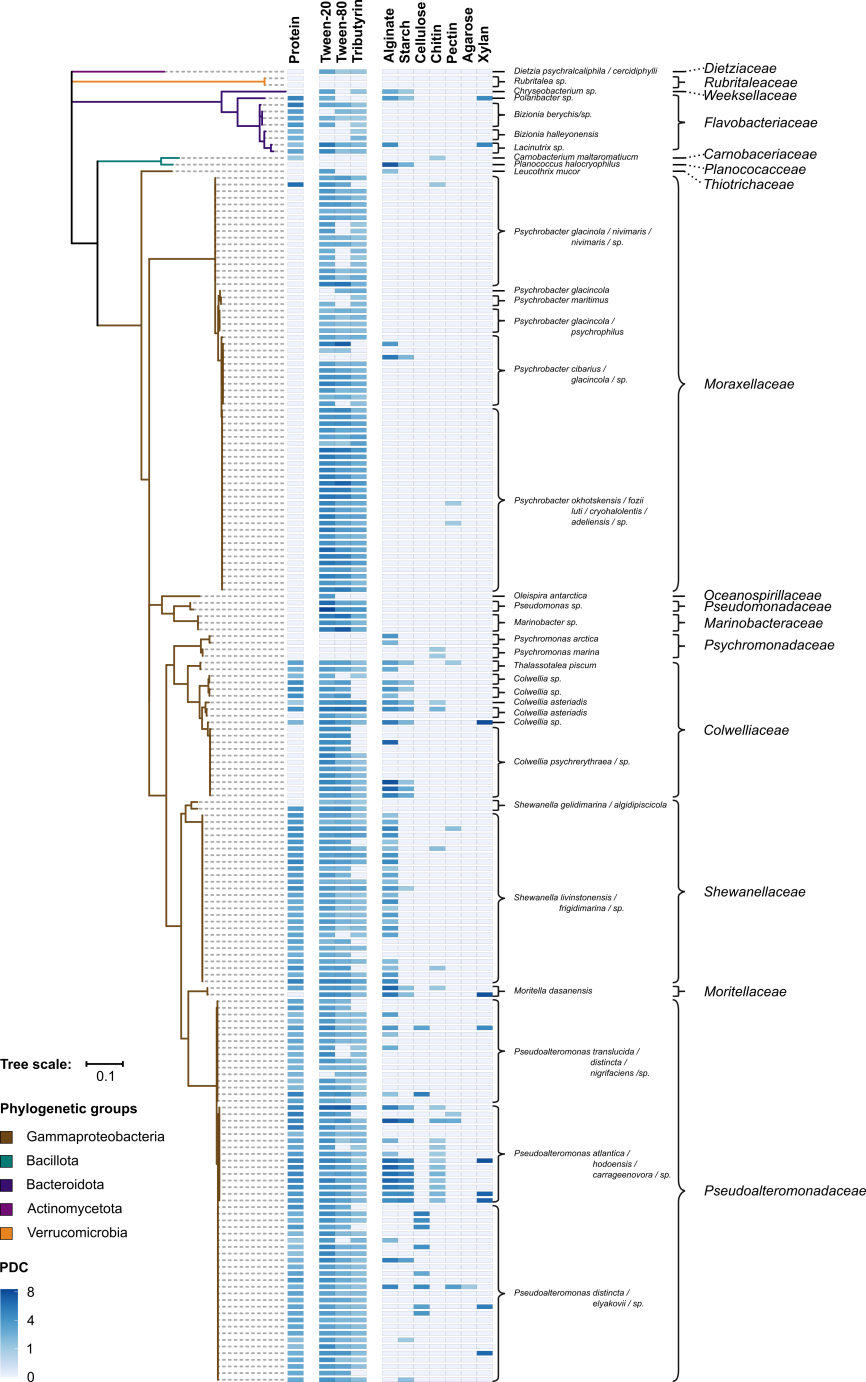
Biopolymer-hydrolysing capacities of 198 pure cultures according to the plate-based assay. Left panel: maximum likelihood phylogenetic tree based on full-length 16S rRNA gene sequences and 1,000 iterations (branch-coloring according to phylogenetic group). Center panel: heat map visualizing the polymer degradation coefficient (PDC) per pure culture and each tested biopolymer. Right panel: for each tested pure culture, the closest related known species and family are indicated.

The pure cultures were studied with plate-based assays designed to cover the various types of ingested phyto- and/or zooplankton-derived biopolymers and their characteristic chemical bonds targeted by hydrolases (Fig. 3). The tested compounds included (i) tributyrin, tween-20, and tween-80 to represent lipid-like polymers harboring carboxyl ester bonds; (ii) casein in the form of skimmed milk powder to represent proteins with their characteristic peptide bonds; and (iii) chitin, starch, xylan, cellulose, alginate, pectin, and agarose to represent natural polysaccharides with α/β-(1-4)- or β-(1-3)-glyosidic bonds. Except for agarose, chemical structures and clearance zones in the plate assays indicative of biopolymer hydrolysis are exemplified in Fig. 3. Biopolymer hydrolysis as revealed by the plate assays were semi-quantitatively evaluated by calculating the polymer degradation coefficient (PDC), i.e., the ratio between the diameters of clearing zones and their central colonies. The biopolymer-specific PDCs of the 198 isolates according to their phylogenetic position are illustrated in Fig. 4.

Notably, the three tested lipids were degraded by most of the tested isolates (~81–92%), albeit with varying PDC levels (1.05–7.5); the most efficient lipid utilization was observed within *Pseudomonas* spp. (PDC up to 7.5), while the genera *Carnobacterium* and *Rubritalea* were apparently not able to utilize these lipids. The second most commonly utilized polymer was casein, degraded by ∼50% of the isolates. Strikingly, taxon-specific patterns of protein degradation were observed, with positive results for nearly all representatives of the genera *Pseudoalteromonas* and *Shewanella* and negative results for, e.g., the genera *Psychrobacter*, and *Psychromonas*, and the phylum *Verrucomicrobia*. Similar observations were previously reported for the krill gut microbiome as well as other marine habitats (38, 39). In general, except for cellulose, agar, and pectin, all tested biopolymer degradation capabilities viewed as a phylogenetic signal showed a significant *P* ≤ 0.005 value and also Blomberg’s *K* was close to zero, suggesting a non-random arrangement. This corroborates the observation from the heat map in Fig. 4 that hydrolytic capacities are often conserved on the level of closely related strains but do not consistently follow higher-level phylogenetic branching.

The greatest potential for biopolymer hydrolysis was observed with isolates affiliating with the genera *Pseudoalteromonas* and *Colwellia* tested for polysaccharides. In accord, *Pseudoalteromonas* spp. are currently recognized as one of the bacterial groups thriving in cold marine habitats and being most proficient for biopolymer (in particular polysaccharide) hydrolysis (39–41). This includes a broad range of hydrolases (chitinase, alginase, amylase, cellulase, xylosidase, agarase, and fucosidase), the composition of which varies across different species (40). The latter is also reflected in the PDC profiles of the here tested *Pseudoalteromonas* spp. (Fig. 4). Similar, albeit less pronounced, hydrolytic capacities were observed with the genera *Colwellia* and *Shewanella* (Fig. 4), which are known to possess biopolymer hydrolases such as alginase, chitinases, and amylases (39, 42–44).

In contrast to these versatile isolates, some were apparently limited to lipid degradation, e.g., *O. antarctica* and *D. psychralcaliphila*, which could reflect a certain nutritional specialization such as on *n*-alkanes (21, 33). In the case of the two Verrucomicrobia isolates, the lack of observable polymer degradation could be due to suboptimal cultivation conditions, since members of this phylum have been implicated in the degradation of methylpentoses during diatom blooms (45).

### Metagenome-derived potential for biopolymer hydrolysis

**Fig 5:**
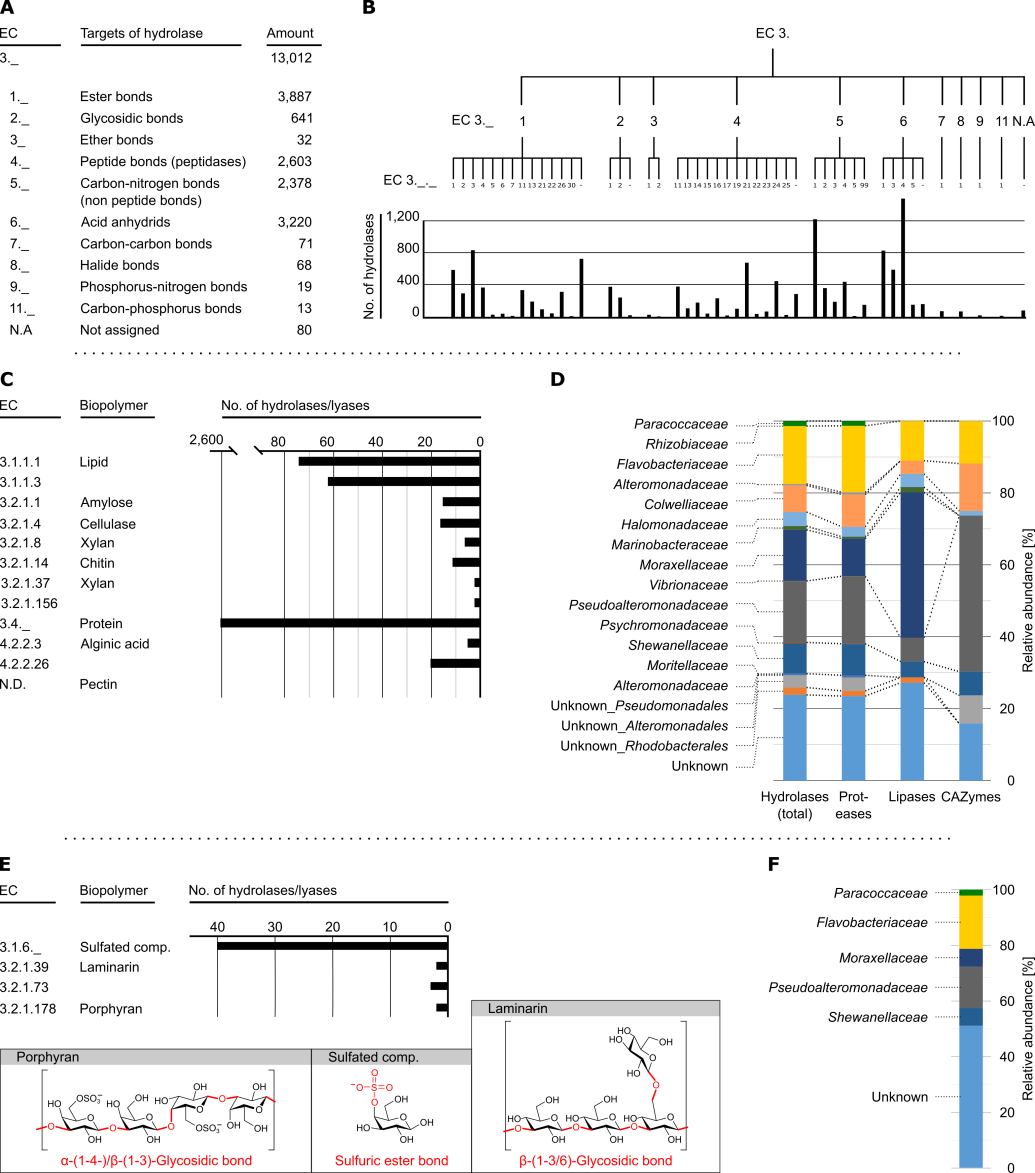
Biopolymer-hydrolysing capacities of cultivated variety according to metagenomic analyses. (**A**) Abundance of predicted hydrolytic enzymes per targeted chemical bond type according to the EC classification. (**B**) Finer resolution at the level of targeted substrate type per chemical bond type. (**C**) Abundance of predicted hydrolases potentially targeting biopolymers investigated by plate-based assays (Fig. 3 and 4). (**D**) Assignment of predicted hydrolase groups (C) to bacterial families. (**E**) Abundance of predicted hydrolytic enzymes targeting phytoplankton-derived biopolymers according to the EC classification. (**F**) Assignment of predicted hydrolase groups (E) to bacterial families.

A metagenomic approach was used to comprehensively assess the potential of the krill gut microbiome for hydrolysis of (bio)polymers. This could unfortunately not be conducted directly with DNA isolated from the gut extract, since it carried a too high load of contaminating host DNA. Therefore, we restricted our analysis to the cultivated microbial variety obtained from aerobically incubated MB plates. A combined Illumina and ONT approach yielded 3,891 contigs (in total 190.43 Mbp), which encode 97,128 predicted proteins including 13,012 hydrolases as predicted by Prokka and InterProScan analyses (Fig. 5).

First, the predicted hydrolases were sorted according to EC numbers (46) allowing a higher-level assignment to the chemical bonds they target (Fig. 5A and B). The vast majority (~96%) of the hydrolases belongs to EC 3.1 (~30%, targeting ester bonds), EC 3.2 (~5%, targeting glycosidic bonds), EC 3.4 (~20%, targeting peptide bonds), EC 3.5 (~18%, targeting non-peptide carbon-nitrogen bonds), and EC 3.6 (~25%, targeting acid anhydrides). This agrees well with food ingested by krill to be dominated by proteins, lipids, and polysaccharides (7).

In consideration of the plate-based assays (Fig. 3 and 4), the metagenomic data set was also screened for hydrolases targeting the biopolymers applied in these assays (Fig. 5C). At first glance, hydrolases targeting lipids (in total 136) and proteins (2,603) dominate the range of attributable hydrolases, which agrees well with the observed broad capacity of the isolates to utilize lipids and proteins (Fig. 4). Also, in accord with the experimental data, polysaccharide-targeting hydrolases are less numerous. In the next step, the distribution of all hydrolases [combined, proteases, lipases, and carbohydrate-active enzymes (CAZymes)] across bacterial families was studied based on the most likely taxonomic assignment of the respective contigs (Fig. 5D). Most conspicuously, ~40% of the 136 lipases affiliate with the family *Moraxellaceae* and ~43% of the 76 CAZymes with the genus *Pseudoalteromonas*, implicating a phylogenetic accumulation of these hydrolases. While the CAZymes are distributed across the least taxonomic diversity, the contrary is observed for the proteases, which furthermore appear more evenly distributed among the different families.

To assess the hydrolytic capacities of the krill gut microbiome towards phytoplankton-derived polysaccharides, which are often sulfated to a high degree (47), the metagenomic database was additionally searched for predicted sulfatases and hydrolases targeting abundant laminarin and porphyran (48)—no evidence for fucoidan- or carrageenans-targeting hydrolases was obtained. While a considerable number (40) of predicted sulfatases was detected, only few (7) of the queried hydrolases were found (Fig. 5E). In contrast to the aforementioned biopolymer-targeting hydrolases about half of these hydrolases could not be taxonomically assigned, while most of the remaining ones belonged to the families *Flavobacteriaceae* and *Pseudoalteromonadaceae* (Fig. 5F). Recent global analysis indicated that the availability of polysaccharide types in a given habitat shape the repertoire of CAZymes possessed by the respective microbiome (49).

Field studies on the near-surface waters in the North Sea, Antarctica, and Arctica revealed a striking correlation between potential algae polysaccharide-degrading members of the classes *Gammaproteobacteria* and *Flavobacteriia* and the abundance of CAZymes (44, 50–53). Despite general variations of the bacterial community across marine systems (54), the genera *Polaribacter*, *Pseudoalteromonas*, *Shewanella*, and *Colwellia* represent ubiquitous degraders of phytoplankton-derived polysaccharides (51, 52). Pure culture studies revealed members of the family *Moraxellaceae* (in particular genus *Psychrobacter*) isolated from Antarctic habitats to harbor cold-adapted lipases [for review, see reference (55)]. These functional insights into specific groups of the marine pelagic microbiome fairly well agree with the krill gut microbes suggested to decompose biopolymers in the present study (Fig. 5D).

### Metagenome-derived potential for plastic hydrolysis

**Fig 6:**
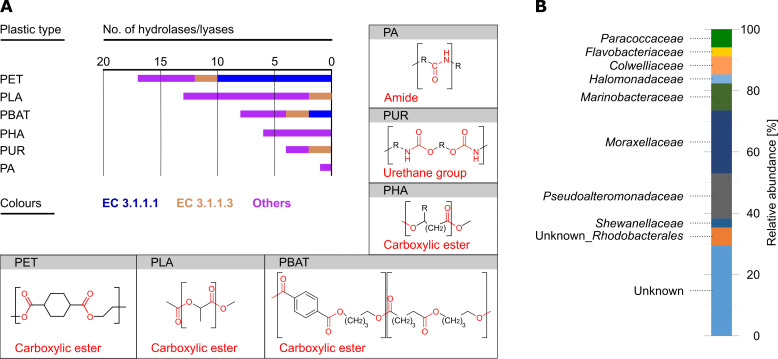
Predicted capacity of cultivated variety to hydrolyze various plastic types according to metagenomic analyses. (**A**) Abundances of hydrolases and their targeted chemical bonds. Abbreviations (alphabetic order): PA, polyamide; PBAT, polybutylene adipate terephthalate; PET, polyethylene terephthalate; PHA, polyhydroxyalkanoate; PLA, polylactide; PUR, polyurethane. (**B**) Assignment of hydrolases to bacterial families.

Considering the increasing prevalence of microplastic across the ocean waters, which also found their way into the Antarctic region (56), the entirety of proteins predicted from the metagenomic data set were searched for homologs of verified plastic decomposing enzymes according to the PAZy database (57). This analysis revealed the potential to hydrolyze the carboxy ester-linked PET (polyethylene terephthalate), PLA (polylactide), PBAT (polybutylene adipate terephthalate), and PHA (polyhydroxyalkanoate), the urethane group containing PUR (polyurethane), and the amide linked PA (polyamide) (Fig. 6A). In total, 34 hydrolases were tentatively assigned to plastic hydrolysis with 9 of them possibly acting on several different plastic types. The majority of hydrolases apparently targets PET and PLA. Notably, in the case of PET, 12 out of 17 hydrolases represent lipases also assigned to lipid degradation (Fig. 5C). Not unexpectedly, the family *Moraxellaceae* provides the largest numbers of putative plastic-hydrolyzing enzymes (Fig. 6B), as noted above for the lipases (Fig. 5D) and also observed in the case of the plate-based assays with the isolates (Fig. 4). Natural plastic-degrading enzymes often comprise functions as lipases, esterases, cutinases or ureases (58, 59), due to the similarity in the backbone of the plastic they can degrade.

### Biopolymer-hydrolyzing potential of gut microbiome versus krill

**Fig 7:**
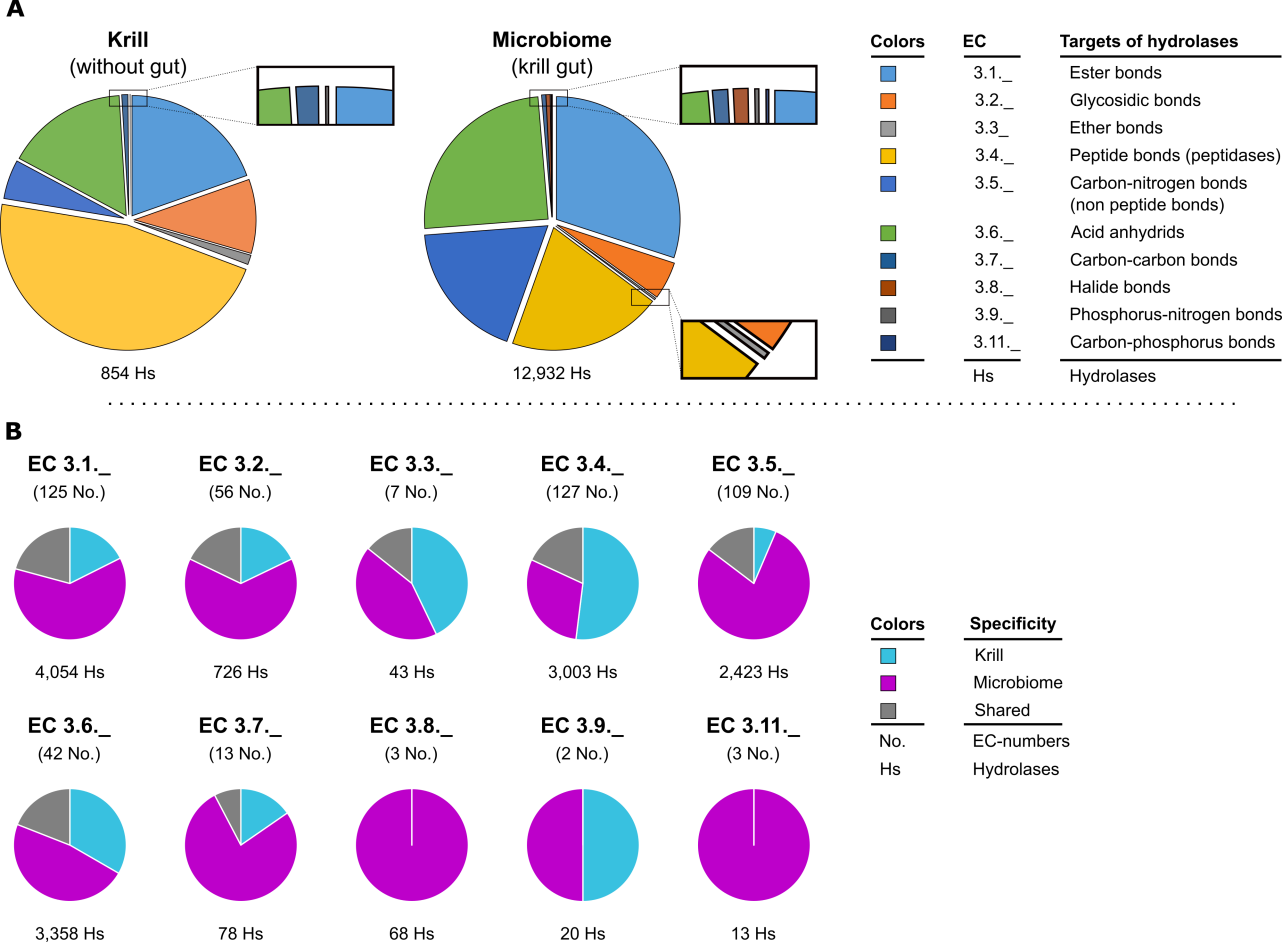
Non-congruent repertoires of hydrolytic enzymes predicted for the cultivated variety of the krill gut microbiome and for its digestive tract-depleted host. (**A**) Abundance of predicted hydrolytic enzymes per targeted chemical bond type according to the EC classification. Data on krill were taken from (10). Data on the microbiome reflect the analysis of the present metagenomic database. (**B**) Finer resolution according to targeted substrate type per chemical bond type differentiating between krill-/microbiome-specific and shared hydrolases. Pie charts reflect the presence/absence of individual hydrolase types only; corresponding pie charts based on determined numbers of hydrolases are shown in Fig. S1. Per targeted chemical bond type (e.g., EC 3.1._), diversity (above pie chart) and abundance (below pie chart) of hydrolases are indicated.

The availability of a transcriptome-proteome compendium of the Antarctic krill depleted of its gut (10) prompted a comparison of the biopolymer-hydrolyzing potentials harbored by the krill animal versus its gut microbiome. Most notably, the aerobically cultivated krill gut microbiome has ~15-fold more different hydrolases at its disposal than its krill host, implicating a prominent role of gut bacteria in processing ingested food. While the hydrolase repertoire of the krill showed a marked bias towards EC 3.4 (~46%, targeting peptide bonds), the vast majority (~91%) of that of the gut microbiome was essentially evenly distributed across the EC numbers 3.1, 3,4, 3.5, and 3.6 (see above section), underlining the broad depolymerizing capacities provided by the gut microbiome (Fig. 7A). This versatility was inspected in more detail according to the different two-digit EC numbers (Fig. 7B). Across all 10 EC numbers, except for EC 3.4. (proteases), the gut microbiome constituted to around 50% or more to the respective sets of hydrolases. The shared hydrolases per two-digit EC number amounted to less than 20%.

*E. superba* is known for its high intrinsic proteolytic activity, even compared to other krill species (e.g., *Meganyctiphanes norvegica*) (60). Therefore, its benefit from microbial proteolytic activities might be negligible. By contrast, krill could take advantage of the multitude of hydrolases targeting, e.g., ester, glycosidic, carbon-nitrogen (non-peptide) bonds, or acid anhydrides (Fig. 7B), provided by the gut microbiome. Thereby, krill could broaden the range of utilizable polysaccharides or increase their digestive efficiency as previously reported for marine isopods (61) or Northern krill (11).

### Conclusions

The present study revealed a high diversity of bacteria in the gut of the Antarctic krill paralleled by an expansive repertoire of hydrolytic enzymes targeting diverse biopolymers as typically present in the food ingested by the krill. This versatility apparently contributes to a large extent to the hydrolytic capacities of the krill holobiont, in particular with respect to ingested polysaccharides. The broad range of biopolymer-degrading enzymes provided by the krill microbiome could benefit the host by enabling an effective breakdown of food, otherwise difficult to degrade or not accessible. The unraveled repertoire of hydrolytic enzymes targeting biopolymers and potentially also plastics represents a treasure chest for biotechnological applications. In this respect, future studies could involve heterologous overproduction and enzymatic characterization of these presumptively cold- and high-salinity-adapted enzymes at a high throughput level.

From an ecophysiological perspective, it would be desirable to extend the metagenomic approach to the gut extract upon depletion of host tissue/DNA to circumvent the cultivation-dependent bias. On the other hand, also cultivation should be extended to cover so far not yet cultivated, but recognized bacterial sub(phyla), including, e.g., *Deltaproteobacteria*, *Epsilonproteobacteria*, *Planctomycetota*, and the enriched *Mycoplasmatota*. Since members of these (sub)phyla comprise (obligate) anaerobs, cultivation strategies must also include anoxic approaches. This would also provide access to the anaerobic members of the Bacteriodota, a phylum observed as quantitatively relevant in the krill gut. Continued investigations into the functional diversity of the krill gut microbiome against the backdrop of the krill’s recently reported genome (48.01 Gbp on chromosome-level) (62), will advance our understanding of the metabolic microbe-host interactions that contribute to the holobiont’s role in the Southern Ocean ecosystem.

During upcoming cruises, four different aspects should be considered: (i) Harvesting of krill should cover different dietary conditions, as these should have shaping effects on the gut microbiome composition. (ii) At the sites of krill sampling, surrounding water bodies should be collected to determine in parallel the microbial diversity in the krill gut and the associated pelagic microbial community. (iii) Temporal and seasonal impacts on the gut microbiota should be covered by investigating krill harvested at different daytimes and seasons. (iv) Developmental stages (juvenile versus adult) and gender of krill may feature differently composed gut microbiota. These endeavors aim to unravel the dynamics of transient and stable microbial communities within the krill gut, opening new directions for comprehensive ecological and biotechnological insights.

## MATERIALS AND METHODS

### Sample collection and krill dissection

Adult krill were sampled between 13^th^ May and 1^st^ June (Antarctic winter) 2021 and 18^th^ January and 7^th^ March (Antarctic summer) 2022 on the Norwegian Krill fishing vessel *Antarctic Endurance*, operating in the Bransfield Strait. Krill swarms were pumped with a vacuum system on a grid in the dewatering room on board the ship. Freshly caught krill was immediately aseptically dissected in a laboratory on the ship. The digestive gut tracts were separately placed in 0.5 mL micro reaction tubes filled with an autoclaved solution of 200 µL glycerol (50%, wt/vol) with 20 mM MgCl_2_ × 6 H_2_O. Tubes were immediately shock frozen in liquid N_2_ and stored at ‒80°C until further processing.

### Krill gut processing

The prepared krill guts were homogenized in marine broth medium (MB) using a 2-mL Kontes Glass dounce tissue disrupters (Kontes Glass—Fischer Scientific, Schwerte, Germany) with gap widths of 76.2–127 µm. The MB medium was prepared by mixing yeast extract (1 g L^‒1^), peptone (8 g L^‒1^), Na_2_HPO_4_ × 2 H_2_O (0.01 g L^‒1^), Na_2_SO_4_ (5.18 g L^‒1^), MgCl_2_ × 6 H_2_O (20.16 g L^‒1^), NaCl (31.12 g L^‒1^), CaCl_2_ × 2 H_2_O (3.81 g L^‒1^), KCl (0.55 g L^‒1^), NaHCO_3_ (0.26 g L^‒1^), and ferric citrate (0.16 g L^‒1^) in deionized water. The pH was then adjusted to 7.6 using 2 M NaOH. After autoclaving (121°C, 20 min) and cooling to 50°C, the MB received 1% (vol/vol) sterile supplement solution. The latter was prepared by mixing KBr (8 g L^‒1^), SrCl × 6 H_2_O (5.72 g L^‒1^), H_3_BO_3_ (2.2 g L^‒1^), Na_2_SiO_3_ × 5 H_2_O (0.7 g L^‒1^), NaF (0.24 g L^‒1^), and NH_4_NO_3_ (0.16 g L^‒1^) in deionized water; after autoclaving the solution was stored at room temperature in the dark.

Preparation of the dounce tissue disrupter involved several steps: (i) The cylindrical glass tube and the pestle were washed once with detergent, followed by several times with deionized water. (ii) Both components were then sterilized in a laminar flow cabinet via submersion in 70% (vol/vol) ethanol for 30 min, followed by air-drying for 15 min. (iii) Prior to use they were rinsed five times with sterile MB.

For breakage of the krill gut, the disrupter glass tube was placed in a rack on ice. Then, the digestive tracts from three to five krill individuals were retrieved from the glycerol storage tubes using sterile tweezers and put together inside the glass tube, followed by the addition of 0.5 mL of ice-cold, sterile MB medium. To avoid loss of material, remaining gut debris in the original tube was sedimented by centrifugation (4,000 × *g*, 10 s, 4°C) and the overlaying glycerol solution was discarded. The debris was resuspended in 0.5 mL ice-cold MB and transferred in the disrupter glass tube as well, resulting in an overall volume of ~1 mL. Then, the pooled krill guts were homogenized by carefully and slowly moving the pestle up and down 10 times. The resulting homogenate was either directly used for cultivation or shock frozen in liquid N_2_ and stored at ‒80°C until molecular analysis.

### Isolation and preservation of bacteria

The isolation of gut bacteria was performed with prepared gut extracts originating from sampling in Antarctic winter (five replicates) and summer (six replicates). For each replicate, homogenates of pooled guts prepared from three to five krill individuals were applied to generate serial dilutions (10^0^, 10^‒1^, and 10^‒2^) in MB. For each of the three dilution steps, 10 parallel MB agar plates (20 g agar L^‒1^) were inoculated with 100 µL dilution by homogenous distribution using a flamed Drigalski spatula. Half of the plates per dilution and replicate each were incubated in the dark at 4°C or room temperature. After incubation for 5‒7 days (at room temperature) and 11‒14 days (at 4°C), respectively, plates were inspected for colony forming units (CFU) and 50‒60 colonies per incubation temperature and sampling season were randomly picked. Selection of colonies was based on morphology, color, and growth behavior to cover the visible diversity as much as possible. Prolonged incubation for up to 6 weeks yielded merely four additional distinct colony types. For purification, picked colonies were streaked onto MB agar plates and incubated under the respective temperature conditions, which were iterated at least five times. Overall, 198 pure cultures were obtained.

For long-term preservation, per pure culture a single colony was picked from the MB agar plate and transferred to 15 mL of MB in a 100-mL Erlenmeyer flask. Cultures were incubated at isolation temperature (room temperature or 4°C) under shaking (60 rpm) for 24 or 72 h, respectively. Then, 0.75 mL of culture broth was mixed with 0.75 mL glycerol (50%, wt/vol) containing 20 mM MgCl_2_ × 6 H_2_O. Stocks were shock-frozen in liquid N_2_ and stored at ‒80°C.

### Plate-based polymer degradation assay

For detection and semi-quantitative analysis of biopolymer degradation, a plate-based assay was used. Isolates were transferred on master plates of MB agar as basal medium supplemented with 1 of 11 different biopolymers (in alphabetic order: agarose, alginate, amylose, casein, cellulose, chitin, pectin, tributyrin, tween-20, tween-80, and xylan). The making of these polymer plates and visualizing of lysis are detailed in the below paragraphs. Plates were incubated at room temperature or 4°C for 5 and 14 days, respectively. Only exception was chitin-containing plates, which were incubated for an extended period of time (15 and 21 days, respectively). Plates were photographed and lysis areas and colony diameters were measured as pixels in the photograph and used to calculate a quotient, which was termed PDC: $PDC=\;\frac{\emptyset \;lysis\;area\;\left[px\right]\;}{\emptyset \;colony\;area\;\left[px\right]}$ . The heatmap displaying all determined PDCs was created via R (63), using the packages “ggplot2” (64) and “tidyverse” (65), and combined with the phylogenetic tree of the tested isolates.

For casein plates, 10% (wt/vol) of skimmed milk powder (composed of >90% of casein) was suspended in deionized water and autoclaved (115°C, 10 min) (66). Then, this skimmed milk suspension was diluted 1:10 with autoclaved, liquid MB agar under constant mixing, prior to pouring into petri dishes. The lysis areas of peptidase-positive isolates are reflected by clear zones in the otherwise turbid agar.

For tributyrin plates, MB agar was supplemented with tributyrin to a final concentration of 1% (vol/vol), followed by autoclaving (120°C, 20 min) (67, 68), cooling to ~50°C under constant stirring and pouring into petri dishes (with continued in-between stirring). Tributyrin-degrading isolates were recognized by clear zones around the colonies in otherwise turbid, fat droplet-containing agar.

For tween-20 or tween-80 plates, the separately autoclaved (120°C, 20 min) detergents (68, 69) were cooled to ~50°C prior to mixing with MB agar to a final concentration of 1% (vol/vol) each and pouring into petri dishes. Tween-20/80-degrading isolates were detected by the formation of white precipitates in the clear agar around the colonies.

For cellulose plates, 1% (wt/vol) carboxymethylcellulose was added to MB agar prior to autoclaving (120°C, 20 min). After cooling to ~50°C under constant stirring, the cellulose-containing MB agar was poured into petri dishes (70, 71). To detect cellulose degradation, plates were flooded for 15 min with a 0.1% (wt/vol) congo red solution (72). Then, the plates were washed two times with 1 M NaCl solution by flooding for 15 min each. Areas of degradation were visible as clear/white areas in the otherwise red-stained agar. For better visualization of clearance zones, stained and washed plates could be flooded with fuming HCl. The drop in pH changes the color from red to blue, which facilitates discrimination between positive and negative isolates (70).

For xylan plates, 0.5% (wt/vol) xylan was added to MB agar prior to autoclaving (120°C, 20 min). After cooling to ~50°C under constant stirring, the xylan-containing MB agar was poured into petri dishes with continued in-between stirring (73). In order to detect areas of xylan degradation, plates were flooded for 15 min with 0.2% (wt/vol) congo red solution. Then, two washing steps (15 min each) with 1 M NaCl followed by likewise flooding the plates. Areas of xylan degradation emerged as either darker or brighter reddish color against the background of a uniform red agar. To improve detection of clearance zones, fuming HCl solution was applied as described above for cellulose plates.

For starch plates, 1% (wt/vol) starch was mixed with MB agar prior to autoclaving (110°C, 21 min). After cooling to ~50°C under constant stirring, the starch-containing MB agar was poured into petri dishes with continued in-between stirring (68). Zones of starch degradation were visualized by flooding the plates with Lugol’s iodine solution, appearing transparent against a deep purple to black background of the agar plates.

For pectin plates, 1% (wt/vol) pectin was mixed with MB agar and adjusted to pH 7.6 prior to autoclaving (120°C, 20 min) (74). After cooling to ~50°C and re-adjusting the pH to 7.6 by adding sterile 2 M NaOH (constant stirring), the pectin-containing MB agar was poured into petri dishes. Zones of pectin degradation were visualized by flooding the plates with Lugol’s iodine solution, appearing transparent against a brown background of the agar plates.

For alginate plates, 1% (wt/vol) alginate was mixed with MB agar prior to autoclaving (120°C, 20 min). After cooling to ~50°C, the alginate-containing MB agar was poured into petri dishes (68). Zones of alginate degradation were visualized by flooding the plates with Lugol’s iodine solution, appearing transparent or differently brownish against a uniform brown background of the agar plates (75).

For chitin plates, colloidal chitin was prepared according to the following procedure (68, 76): To begin with, 15 g of powder shrimp shell chitin was stirred for 60 min at room temperature in 150 mL of fuming (37%) HCl (in 1 L glass beaker) yielding a brownish semifluid. The latter was slowly added to 1,000 mL of ice-cold deionized water under continuous, strong stirring. Residual chitin semifluid was recovered by rinsing the 1 L beaker with 500 mL ice-cold deionized water. The combined 1,500 mL of chitin semifluid was stirred for 10 min and then incubated overnight at 4°C without stirring to allow precipitation. Then, the transparent overlying phase was removed and the remaining turbid suspension was centrifuged (17,700 × *g*, 30 min, 4°C). After discarding the supernatant, the chitin pellet was resuspended in 400 mL deionized water and centrifuged again; this washing step was repeated four times. The washed chitin suspension was adjusted to pH 7 and filtered through paper filters (Rotilabo-folded filters type 600P; Carl Roth GmbH + Co. KG, Karlsruhe, Germany). The chitin-covered filters were placed in ethanol-cleaned petri dishes, dried at 80°C for 3 days, allowing to collect and store (at 4°C) the colloidal chitin. For application to the plate assays, the colloidal chitin was powdered by bead beating: 0.5 g colloidal chitin was placed together with six ceramic beads (three each of 2- and 5-mm diameter) in a 2-mL beating tube and repeatedly shaken at 7.5 m s^‒1^ for 40 s (FastPrep-24 5G; MP Biomedicals, Inc.—Fischer Scientific), yielding a homogenous powder. In preparation for making the chitin plates, 1% (wt/vol) of powdered colloidal chitin were mixed with MB (devoid of peptone and yeast) and 1% (wt/vol) agar prior to autoclaving (120°C, 20 min) (68). Then, approx. 5 mL of this chitin-containing soft agar was layed over the usual solid MB agar and stored at least 24 h prior to use. Zones of chitin degradation appeared transparent against the turbid background of the agar plates.

For agarose plates, the usual MB plates were used. Zones of agarose degradation were detected by sinking of the colonies into MB agar or by softening it below and surrounding the colonies.

### Cultivatable variety

The cultivatable variety complemented our metagenomic analyses. Initially, 12 pooled gut extracts from 36 krill individuals, which proportionally represent Antarctic winter and summer (details provided in Fig. S2), were each split into a ⅓- and a ⅔-aliquot. The ⅓-aliquots were directly used for 16S rRNA gene sequencing. By contrast, the ⅔-aliquots were each spread with a Drigaslki spatula on two MB agar plates, which were incubated for 5 days (room temperature) and 11 days (4°C), respectively (details provided in Fig. S2). Then, the entire microbial mass per plate was scrapped off and transferred each to a 1.5-mL micro reaction tube, followed by shock freezing in liquid N_2_ and storage at ‒80°C until DNA extraction for metagenomic analysis.

### Phylogeny of pure cultures

The phylogenetic affiliations of the isolated pure cultures were determined based on the full-length 16S rRNA gene sequences. For this purpose, the HotSHOT (hot sodium hydroxide and TRIS) extraction method (77) was applied, including a basic lysis buffer (25 mM NaOH, 0.2 mM EDTA, pH 12) and a neutralization buffer (40 mM Tris/HCl, pH 5). For DNA extraction, cell material was scraped off a freshly grown MB agar plate using sterile toothpick and transferred into 75 µL lysis buffer in a 0.2-mL PCR reaction tube. The tube was heated in a thermocycler to 95°C for 20 min and then cooled to 4°C. Subsequently, 75 µL of neutralization buffer was added to the mixture and followed by gentle mixing. The resulting DNA extract was stored at ‒20°C.

Full-length 16S rRNA gene-sequencing was performed by LGC Genomics GmbH (Berlin, Germany) using the universal bacterial primers 27f, 1492r (78). Obtained sequences were quality checked, trimmed, and aligned using FinchTV and SNAPGene (www.SNAPGene.com). Initial identification of the closest relative was achieved with BLAST (79) applying the standard settings with selecting hits < 1.0*E−20 and >97% sequence identity. In each case, the closest sequence hit with the highest score and full species name was used as identifier. If multiple species entries result in an identical score, both names are stated. If full species name could not be provided isolates were named at genus level and the species identifier “sp.” An additional quality check was based on the NCBI implementation of Uchime, which enables the identification of chimeras deviating >3% from the closest parent.

For the generation of a phylogenetic tree, the full-length 16S rRNA gene sequences were aligned using the program MEGA (v.11) (80) with ClustalW (81) in standard settings. The aligned sequences were then trimmed to a maximal consensus area covering all isolates. The Intern modeling program from MEGA was used for the maximum likelihood model, with K2 + G + I resulting in the lowest BIC score. The generated maximum likelihood tree was based on 1,000 iterations, uploaded in iTOL (82), and graphically modified with InkScape version 1.0.1 (https://inkscape.org/).

In order to test the correlation between phylogenetic branching and the determined hydrolytic capacities (calculated PDC values) of each tested strain, the package “picante” was used to calculate the Blomberg’s *K* value and PIC variance P (83).

### DNA extraction

For DNA extraction, 12 gut extracts and 24 cultivation samples (see Fig. S2. and above section Cultivatable variety) were used. For both sample types, two different DNA extraction approaches were applied according to the manufacturer’s recommendations: ZymoBIOMICS DNA Miniprep KIT (Zymo Research Corp., Irvine, CA, USA) and MagAttract HMW DNA Kit (Qiagen, Hilden, Germany). For purification, the resulting DNA extracts were mixed with AMPure XP beads in a 1:1 ratio (vol/vol) and treated according to the manufacturer’s protocol (Beckman Coulter, Brea, CA, USA). Quality control and concentration measurements of isolated DNA were done using the Fragment Analyzer instrument (Agilent Technologies, Santa Clara, CA, USA).

### Full-length 16S rRNA gene amplicon sequencing and taxonomic classification

Purified DNA extracts (10 ng in 10 µL elution buffer) obtained with the ZymoBIOMICS approach were barcoded using the 16S Barcoding Kit 1–24 (SQK-16S024; ONT, Oxford, UK). Per sample, the complete 10 µL barcoded DNA was loaded on a MinION Flow Cell R9.4.1 (ONT), followed by sequencing for 72 h using the MiniKNOW software (ONT). The resulting raw reads were base-called and demultiplexed by using ONT’s Guppy basecaller (84). Following the quality-check of remaining reads with NanoPlot (85), only those with a *Q*-score >9 and a length of 1–2 kbp were continued to be used and yielded in total 2,134,365 16S rRNA gene sequences (N50 = 1.58 kbp) for the gut extracts and 1,854,554 (N50 = 1.56 kbp) for the cultivated variety.

These 16S rRNA gene sequences were uploaded to ONT’s Epi2ME 16S pipeline (86) for taxonomic classification searching against the NCBI Taxonomy database (87) with BLASTn (79). Hits were accepted on the species level according to the following standard settings implemented in the Epi2ME pipeline for nanopore sequences and similar to thresholds previously applied (88): *e*-value <0.01, coverage ≥60%, identity ≥77%, and max. target sequences equal to 3. In total, 2,116,085 (av. accuracy 89%) and 1,846,821 (av. accuracy 93%) full-length 16S rRNA gene sequences were obtained from the gut extracts and cultivated variety, respectively. Assigned species with <3 reads in the total data sets were excluded which led to finally considered read numbers of 2,078,787 and 1,788,289 for gut extracts and cultivated variety, respectively, taxonomically assigned to a total of 2,309 bacterial species. For the most abundant species in each family found, the reference 16S rRNA gene sequence identical to its taxonomic identifier was downloaded from the NCBI Taxonomy database (87) using a custom Python script, utilizing the Entrez Programming Utilities API (www.ncbi.nlm.nih.gov/books/NBK25501/). The sequences served as basis for calculating and constructing the phylogenetic tree as described above in the section “Phylogeny of pure cultures.”

### Metagenome sequencing

Initial attempts to access the metagenome of the microbiome directly from prepared guts were impeded by interfering host tissue. Therefore, we focused on the cultivated variety for this purpose. To enhance coverage of functional diversity four different settings of the cultivated variety from gut extracts were investigated, i.e., Arctic summer and winter seasons, and for each of them plate incubations at room temperature and 4°C. A detailed scheme integrating tested conditions, plate cultivations, and type of DNA extractions is presented in Fig. S2. Three different DNA extracts were analyzed: (i) room temperature and (ii) 4°C incubations each combined from ZymoBIOMICS-extractions, (iii) room temperature and 4°C incubations pooled after MagAttract-extraction. All three DNA extracts were subjected to metagenomic sequencing combining short-read (Illumina) and long-read (ONT nanopore) approaches (Fig. S3A).

For the short-read whole-genome shotgun sequencing (WGS), 1 ng of DNA per DNA extract was used to generate extract-specific Illumina libraries by applying the Nextera XT DNA Library Preparation Kit (Illumina, San Diego, CA, USA). In the case of the pooled MagAttract DNA extract, two technical replicates were run. The four libraries were sequenced on the Illumina NextSeq 2000 platform (Illumina), applying 2 × 50 bp (DNA extracts with ZymoBIOMICS) and 2 × 300 bp (DNA extracts with MagAttract HMW) configurations. Resulting Illumina sequence data were processed via three steps (Fig. S3B, upper panel): (i) demultiplexing was performed with the bcl2fastq2 (v2.20.0.422) software using the standard settings (Illumina); (ii) quality checking of sequences was performed using FastQC (89); (iii) adapters were removed, quality trimmed, and size filtered by using BBDuk from the BBTools package (http://jgi.doe.gov/data-and-tools/bb-tools/), resulting in the exclusion of reads with *Q*-scores <20 and lengths <20 bp (for 2 × 50 bp libraries) or <50 bp (for 2 × 300 bp libraries) (90).

For long-read sequencing, 1 µg per DNA extract was used. ZymoBIOMIC- versus MagAttract-based DNA extracts were prepared with the Ligation Sequencing kits LSQ-LSK109 versus 114 (ONT), with the LIG-approach reported as most suitable for bacterial genomes (91). Again, two technical replicates were run from the pooled MagAttract DNA extract. The generated LIG libraries were then loaded on the MinION Flow Cells R9.4.1 versus R10.4.1 and sequenced for 48 versus 72 h using MinKNOW (ONT). Resulting raw reads were base-called with Dorado (v.0.3.1; ONT) and demultiplexed by using Guppy (ONT) (84). In both cases, high-accuracy base-calling was performed on the GPU-enabled server operated with Rocky Linux OS. Quality-check with NanoPlot (85) was performed before and after trimming. Adapter removal was conducted with PoreChop (92). Trimming and filtering were carried out with NanoFilt (ONT) to remove reads <1 kbp and *Q*-score <15 (85).

### Hybrid metagenomic assembly integrating taxonomic/functional annotation

For hybrid assembly, the pipeline (meta)SPADES (93, 94) was used to incorporate all filtered Illumina and ONT reads (Fig. S3B, lower panel). Draft assemblies were filtered by a custom script to remove contigs with lengths <5–10 kbp and coverages <10.0. A two-fold polishing by means of ONT reads followed by Illumina reads was achieved with NanoPolish (95) and Racon (96), respectively. Then functional analyses and taxonomical classification (see paragraphs below) were performed. The quality control of the metagenomic assemblies was performed with QUAST (97) at each step (including draft, primary, and final state).

For functional analysis, the generated metagenomic assemblies were analyzed with Prokka (98) to identify and annotate genes. This initial annotation was refined by analysis with InterProScan (99). A custom tool written in Perl (100) was employed to integrate both lines of functional annotation along with the taxonomic information (see paragraph below).

For taxonomic classification, the assembled contigs were analyzed with the MetaWRAP classification module (101) in a two-step process: (i) MegaBLAST (102) was used to align each individual contig against the NCBI_nt database (103) and (ii) the results of the alignment were used by the *Taxator-tk* software to perform the taxonomic assignment for each contig (104).

### Bioinformatic prediction of hydrolytic enzymes

The metagenomic data set was searched for predicted, biopolymer-targeting hydrolytic enzymes via several iterative steps: (i) initially, a comprehensive survey was conducted by detecting predicted enzymes, which affiliate with the hydrolase-typifying EC number 3._ and all its hierarchical sub-levels, as previously reported for the krill tissue (10). This was achieved with a custom script written in R (63). (ii) For comparison with the plate-based assays, a specific search was conducted for predicted hydrolases targeting the 10 experimentally investigated biopolymers. In case of alginate, the respective ligases involved in its degradation were considered. (iii) To include typical phytoplankton-derived biopolymers, also hydrolases targeting sulfated polymers, laminarin, and porphyran were searched for according to their specific EC numbers.

For the detection of potential plastic-degrading enzymes, the database “PAZy – The Plastics-Active Enzyme Database” was used (57). The extracted PAZys were queried against the generated metagenomic data set using BLASTp (79) and applying the following settings: identities ≥40%, sequence alignment ≥100 nt, *e*-value ≤0.1E−10, and bit-score ≥100.

### Graphics

Graphics were done with Inkscape version 1.0.1 (https://inkscape.org/). Drawings of chemical structures were created using ChemDraw Professional version 22.0.0.22 (Revvity Signals Software Inc., Waltham, MA, USA). In case of Fig. 1 and Fig. S3, the BioRender package was used (BioRender.com). Taxonomic trees were visualized and modified using the web-based program iTOL (82).

## Data Availability

All data this study builds on are presented in the manuscript, the supplemental material, and under the Genbank accession numbers and Github links: PRJNA1028309 (full-length 16S rRNA gene sequences of pure cultures determined by the Sanger sequencing method), PRJNA1028309 (full-length 16S rRNA gene sequences from the gut extract determined by the ONT method), https://github.com/homeveg/KiGuMi/tree/main/data (hydrolase sequences assembled from Illumina and ONT metagenomic sequencing), and https://github.com/homeveg/KiGuMi/tree/main/data (contig sequences harboring the analyzed hydrolase encoding genes).

## SUPPLEMENTAL MATERIAL

Fig. S1 spectrum.04035-23-s0001.tiff Abundances of hydrolases from krill vs its gut microbiome.

Fig. S2 spectrum.04035-23-s0002.tiff Generation of DNA extracts for metagenomics.

Fig. S3 spectrum.04035-23-s0003.tiff Workflow of NGS procedure.

Table S1 spectrum.04035-23-s0004.xlsx Data from the plate-based biopolymer degradation assays.

Table S2 spectrum.04035-23-s0005.xlsx Predicted hydrolytic enzymes underlying Fig. 5AB.

Table S3 spectrum.04035-23-s0006.xlsx Predicted hydrolytic enzymes underlying Fig. 5CD.

Table S4 spectrum.04035-23-s0007.xlsx Predicted hydrolytic enzymes underlying Fig. 5EF.

Table S5 spectrum.04035-23-s0008.xlsx Predicted hydrolytic enzymes underlying Fig. 6AB.

Table S6 spectrum.04035-23-s0009.xlsx BLAST results of 16S rRNA genes of isolates.

ASM does not own the copyrights to Supplemental Material that may be linked to, or accessed through, an article. The authors have granted ASM a non-exclusive, world-wide license to publish the Supplemental Material files. Please contact the corresponding author directly for reuse.
