# Supplementary figures and images for: Rich microbial and depolymerising diversity in Antarctic krill gut
Source: Microbiol Spectr. 2024 Mar 11;12(4):e04035-23. doi: 10.1128/spectrum.04035-23 (PMC10986584; doi:10.1128/spectrum.04035-23)

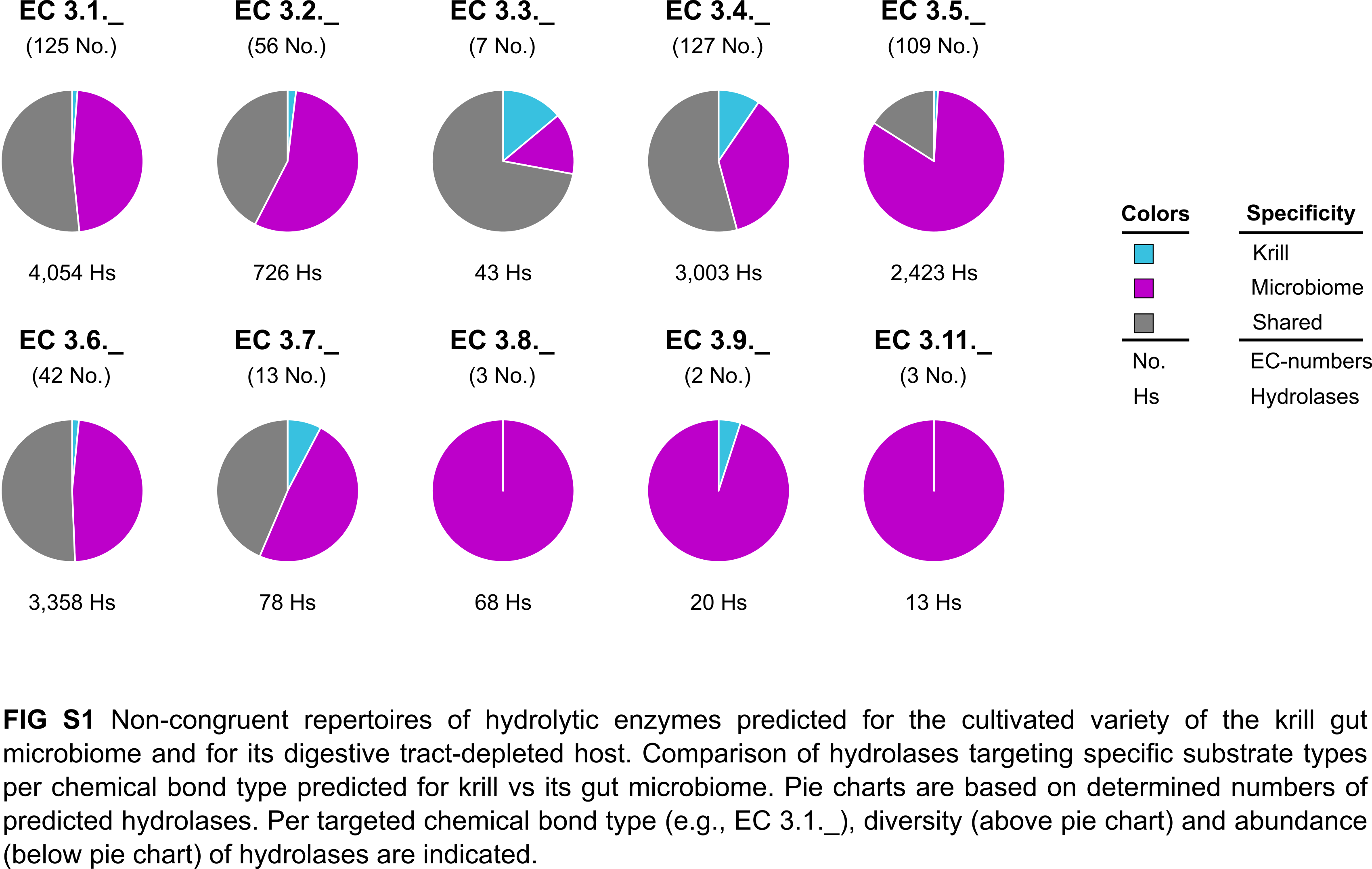

Supplement: Fig. S1 — Abundances of hydrolases from krill vs its gut microbiome. [file spectrum.04035-23-s0001.tiff]

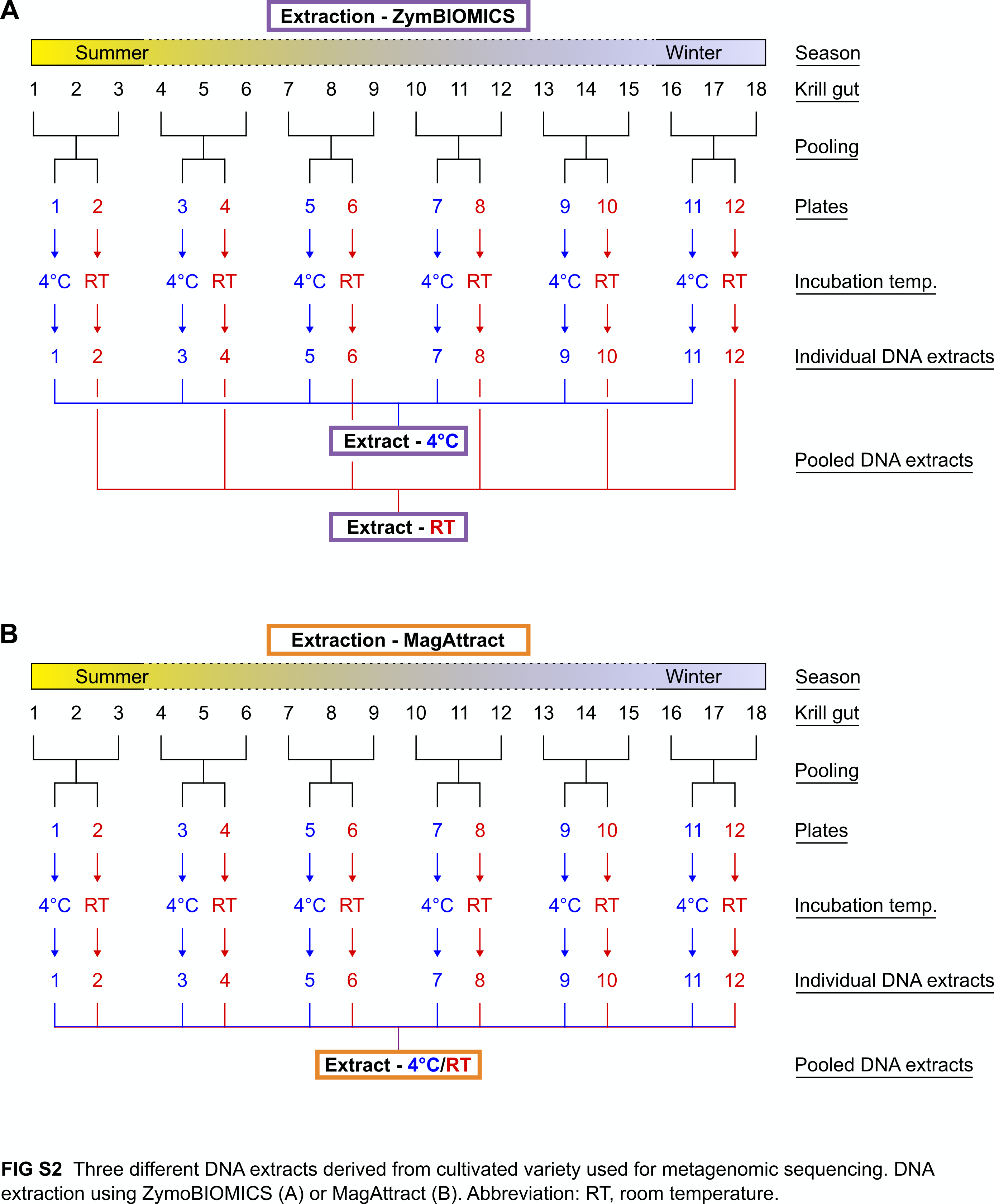

Supplement: Fig. S2 — Generation of DNA extracts for metagenomics. [file spectrum.04035-23-s0002.tiff]

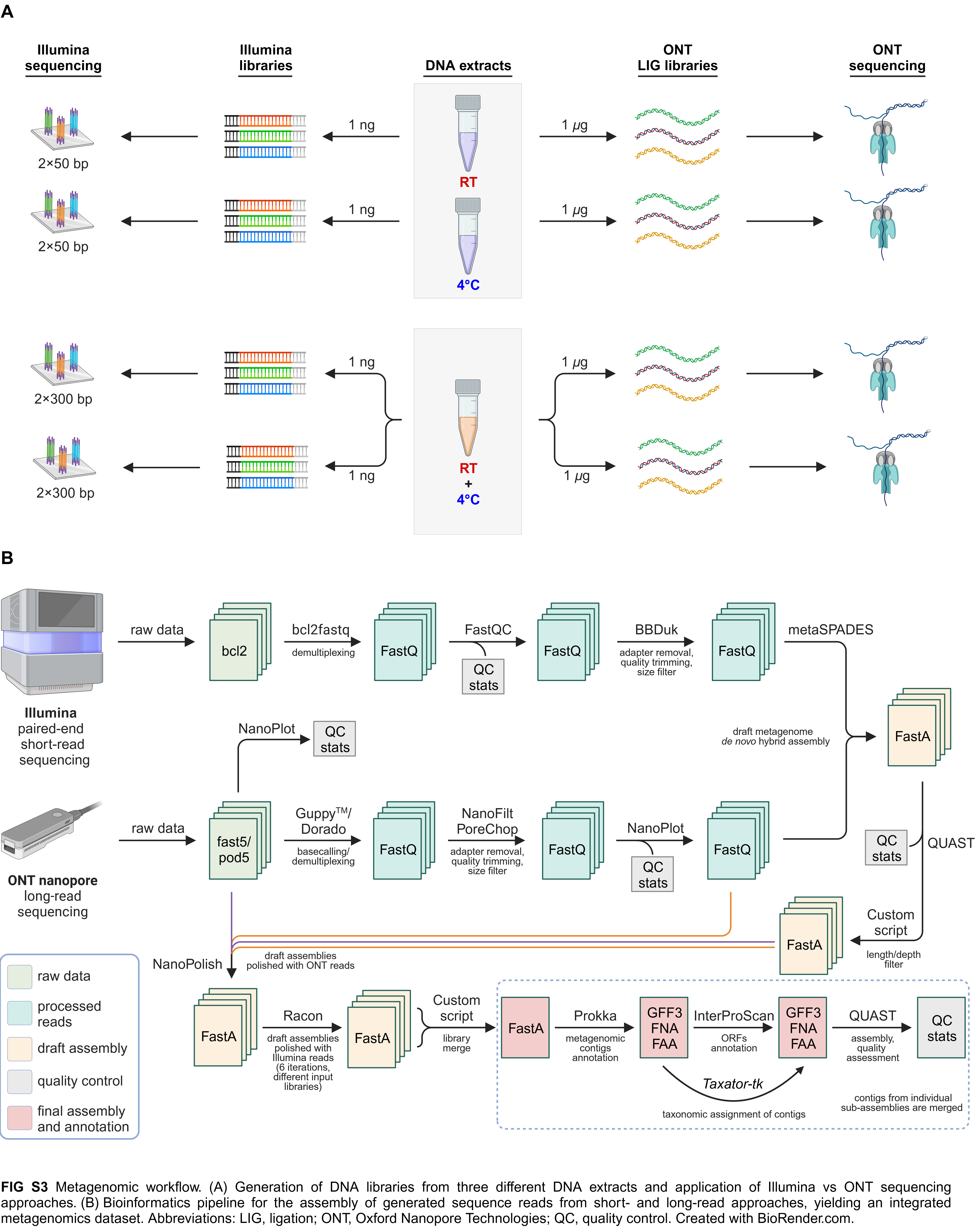

Supplement: Fig. S3 — Workflow of NGS procedure. [file spectrum.04035-23-s0003.tiff]
